# Acute kidney injury and COVID-19 in Brazil: disparities in outcomes between public and private hospitals in Rio de Janeiro reveal issues of equity in intensive care

**DOI:** 10.1590/2175-8239-JBN-2026-E007en

**Published:** 2026-05-25

**Authors:** Lorruan Alves dos Santos, Emmanuel de Almeida Burdmann, Luis Yu

**Affiliations:** 1 Universidade de São Paulo, Departamento de Nefrologia, São Paulo, SP, Brazil.

Acute kidney injury (AKI) is a frequent and lethal complication in patients with moderate to severe COVID-19. Literature reviews with meta-analyses estimate the incidence of AKI to be between 32.6% and 46% in patients admitted to intensive care units (ICUs) with COVID-19^1,2^. This variation reflects differences in diagnostic criteria, the severity of included patients, and, not insignificantly, the availability of healthcare resources. In cohorts of ICU patients with COVID-19, overall mortality is approximately 25%, but rises to 47–52% when AKI is present^1,3^. In Brazil, a country characterized by a dual healthcare system (public and private), comparative literature between the two sectors regarding AKI outcomes is scarce and contradictory^4^.

The study by Faria et al.^5^, published in the Brazilian Journal of Nephrology, provides a relevant contribution. It is a retrospective cohort study including 2,333 patients admitted to ICUs of one public and one private hospital in Rio de Janeiro between March 2020 and April 2022. The authors used standardized criteria (KDIGO – Kidney Disease: Improving Global Outcomes e ADQI – Acute Disease Quality Initiative) to classify AKI and acute kidney disease, in addition to robust statistical modeling, including multivariate logistic regression, Cox models, and the Fine–Gray competing risks model to analyze renal recovery.

The study results are both expected and concerning. Despite the high and similar incidence of AKI in both settings (80.4% in the private hospital versus 78.8% in the public hospital; p = 0.315)—values higher than those estimated in international meta-analyses—the main outcomes differed significantly^1,2^. Overall mortality was higher in the public hospital (46.7% versus 31.3%; p < 0.001), and admission to this service was an independent predictor of AKI (OR 1.279; 95% CI: 1.012–1.620) and in-hospital mortality (HR 1.675; 95% CI: 1.435–1.956), even after adjustment for age, clinical severity, and mechanical ventilation. These results contrast with previous data from Gomes et al.^4^, which found no influence of hospital type on survival among patients with dialysis-requiring AKI in Brazil in a pre-pandemic context, suggesting that COVID-19 may have amplified previously attenuated disparities.

Conventional logistic regression suggested apparently higher renal recovery rates in the public hospital, which is counterintuitive for those familiar, at least in part, with the challenges of Brazil’s public healthcare system. However, one of the methodological strengths of the present study lies in the application of the Fine–Gray model. By treating death as a competing event, the model revealed that the probability of recovery was significantly lower in the public sector (SHR 0.650; 95% CI: 0.554–0.762; p < 0.001). The statistical rigor and critical stance of the authors are instructive and reinforce the importance of analytical approaches that account for the data structure in hospital-based studies, in which high mortality may mask the true recovery trajectory among survivors.

Differences in the availability of therapeutic resources across hospitals help explain the study findings. In the private hospital, there was greater access to continuous renal replacement therapy (CRRT), prolonged intermittent therapy, extracorporeal membrane oxygenation, noninvasive ventilation with a high-flow nasal cannula, and tracheostomies. In the public hospital, intermittent hemodialysis accounted for 94.6% of modalities, and CRRT was not available. Multinational studies have already shown that low- and middle-income settings have significantly higher mortality rates from AKI associated with COVID-19, even after adjusting for clinical severity, reinforcing the role of resource availability as a determinant of outcomes^6^.

Some limitations warrant caution in interpreting the results. The retrospective design and restriction to only two hospitals in Rio de Janeiro limit generalizability. The lack of complete data on race and body mass index prevented inclusion of these variables in the models, representing a relevant gap given the role of social determinants in COVID-19 outcomes and kidney health^4,7,8^. The use of the lowest creatinine level as the baseline value during hospitalization, although widely used, may overestimate AKI incidence and hinder comparisons between studies^9^. The absence of post-discharge follow-up precludes evaluation of the impact of AKI on long-term outcomes, an important dimension in the context of long COVID^10^. The scarcity of comparative studies between public and private sectors in addressing COVID-19 in both national and international literature limits the possibility of situating these findings within a broader evidence base.

Despite these limitations, the findings by Faria et al.^5^ point to a conclusion that extends beyond nephrology: equity in access to intensive care resources is a structural determinant of health outcomes. The pandemic, by overburdening already fragile systems (not only healthcare), deepened preexisting inequalities and demonstrated that the same clinical condition may result in very different outcomes depending on the care context. Therefore, investing in the strengthening of public ICUs, with expanded access to advanced renal and multiorgan support modalities, should not be considered a temporary response to health crises, but as a state policy aimed at reducing health inequities in Brazil. In this sense, more than documenting these disparities, it is necessary to translate this knowledge into concrete actions. The field of Social and Human Sciences in health, one of the structural pillars of public health, has much to contribute to this discussion.
