# Supplementary material for: Acute kidney injury and COVID-19 in Brazil: disparities in outcomes between public and private hospitals in Rio de Janeiro reveal issues of equity in intensive care
Source: J Bras Nefrol. 2026 May 25;48(2):e2026E007. doi: 10.1590/2175-8239-JBN-2026-E007en (PMC13233051; doi:10.1590/2175-8239-JBN-2026-E007en)
Supplement: Translation

# Translation: Injúria renal aguda e COVID-19 no Brasil: desigualdades nos resultados entre hospitais públicos e privados do Rio de Janeiro revelam questões de equidade no atendimento intensivo

**DOI:** 10.1590/2175-8239-JBN-2026-E007pt

**Authors:** Lorruan Alves dos Santos, Emmanuel de Almeida Burdmann, Luis Yu

**Affiliations:** 1 Universidade de São Paulo, Departamento de Nefrologia, São Paulo, SP, Brasil.

A injúria renal aguda (IRA) é uma complicação frequente e letal em pacientes com COVID-19 moderada ou grave. Revisões de literatura com metanálises estimam a incidência de IRA entre 32,6% e 46% em pacientes internados em unidades de terapia intensiva (UTI) com COVID-19^1,2^. Essa variação reflete as diferenças nos critérios diagnósticos empregados, na gravidade dos pacientes incluídos e, de forma não negligenciável, na disponibilidade de recursos assistenciais. Em coortes de pacientes com COVID-19 na UTI, a mortalidade global é de aproximadamente 25%, mas sobe para 47–52% quando há IRA associada^1,3^. No Brasil, país caracterizado por um sistema de saúde dual (público e privado), a literatura comparativa entre os dois setores quanto aos desfechos da IRA é escassa e contraditória^4^.

O estudo de Faria et al.^5^, publicado no *Brazilian Journal of Nephrology*, oferece contribuições relevantes. Tratase de um estudo de coorte retrospectivo com 2.333 pacientes internados em UTIs de um hospital público e de um hospital privado no Rio de Janeiro, entre março de 2020 e abril de 2022. Os autores empregaram critérios padronizados (KDIGO – *Kidney Disease: Improving Global Outcomes* e ADQI – *Acute Disease Quality Initiative*) para classificação de IRA e de doença renal aguda, além de modelagem estatística robusta, incluindo regressão logística multivariada, modelos de Cox e o modelo de riscos competitivos de *Fine-Gray* para análise da recuperação renal.

O resultado do estudo é previsível e preocupante, pois, a despeito da incidência de IRA ter sido elevada e semelhante nos dois contextos (80,4% no privado vs. 78,8% no público; p = 0,315), estes valores são superiores à frequência estimada em metanálises internacionais^1,2^, e os principais desfechos analisados divergiram significativamente. A mortalidade geral foi maior no hospital público (46,7% vs. 31,3%; p < 0,001), e a admissão nesse serviço foi um preditor independente de IRA (OR 1,279; IC95%: 1,012–1,620) e de mortalidade intrahospitalar (HR 1,675; IC95%: 1,435–1,956), mesmo após ajuste para idade, gravidade clínica e ventilação mecânica. Esses resultados contrastam com dados anteriores de Gomes et al.^4^, que não identificaram influência do tipo de hospital na sobrevida de pacientes com IRA dialítica no Brasil, em um contexto pré-pandêmico, sugerindo que a COVID-19 pode ter amplificado disparidades previamente atenuadas.

A regressão logística convencional utilizada sugeria taxas de recuperação renal aparentemente maiores no hospital público, o que é contraintuitivo para quem conhece, ao menos em parte, as mazelas e os desafios do sistema público de saúde no Brasil. Contudo, um dos méritos metodológicos do presente estudo reside na aplicação do modelo de *Fine-Gray*, uma vez que o tratamento do óbito como evento competitivo revelou que a probabilidade de recuperação era significativamente menor no setor público (SHR 0,650; IC95%: 0,554–0,762; p < 0,001). O rigor estatístico e a postura crítica dos autores são didáticos e reforçam a importância de abordagens analíticas que contemplem a estrutura dos dados em estudos hospitalares, nos quais a elevada mortalidade pode mascarar a verdadeira trajetória de recuperação dos sobreviventes.

A diferença na disponibilidade de recursos terapêuticos acessíveis às equipes hospitalares corrobora a explicação dos achados do estudo. No hospital privado, houve maior acesso à terapia de substituição renal contínua (TSRC) e à terapia prolongada intermitente, à oxigenação por membrana extracorpórea, à ventilação não invasiva com cânula nasal de alto fluxo e às traqueostomias. No hospital público, a hemodiálise intermitente correspondeu a 94,6% das modalidades, e a TSRC não estava disponível. Estudos multinacionais já demonstraram que contextos de baixa e média renda apresentam taxas de mortalidade por IRA associada à COVID-19 significativamente superiores, mesmo após ajuste para gravidade clínica, o que reforça o papel da disponibilidade de recursos como determinante dos desfechos^6^.

Algumas limitações impõem cautela na interpretação dos resultados. A natureza retrospectiva e a restrição a somente dois hospitais da cidade do Rio de Janeiro limitam a generalização dos achados. A ausência de dados completos sobre raça e índice de massa corporal impediu a inclusão dessas variáveis nos modelos, o que constitui uma lacuna relevante, dado o papel dos determinantes sociais em desfechos de COVID-19 e na saúde renal^4,7,8^. A utilização da creatinina mais baixa como valor basal durante a internação, embora amplamente empregada, pode superestimar a incidência de IRA e dificultar comparações entre estudos^9^. A ausência de seguimento pós-alta impede a avaliação do impacto da IRA sobre desfechos em longo prazo, dimensão relevante no contexto da COVID-19 longa^10^. A escassez de estudos comparativos entre os setores público e privado no enfrentamento da COVID-19 na literatura nacional e internacional limita a possibilidade de confrontar os presentes achados em um contexto mais amplo de evidências científicas.

Apesar dessas limitações, os dados de Faria et al.^5^ apontam para uma conclusão que ultrapassa o campo de estudos em nefrologia: a equidade no acesso a recursos de terapia intensiva é um determinante estrutural dos desfechos em saúde. A pandemia, ao sobrecarregar sistemas (não só o de saúde) já fragilizados, aprofundou desigualdades pré-existentes e demonstrou que a mesma condição clínica pode resultar em desfechos muito diferentes, a depender do contexto assistencial. Dessa forma, investir na capacitação de UTIs públicas, com ampliação do acesso às modalidades avançadas de suporte renal e de outros órgãos, não deve ser entendido como uma resposta pontual a crises sanitárias, mas como uma política de Estado voltada à redução das iniquidades em saúde no Brasil. Nesse sentido, mais do que documentar essas disparidades, é preciso converter esses conhecimentos em ações concretas. Para isso, o campo das Ciências Sociais e Humanas em saúde, um dos pilares estruturais da saúde coletiva, tem muito a contribuir para essa discussão.
